# Sperm Global DNA Methylation (SGDM) in Semen of Healthy Dogs

**DOI:** 10.3390/vetsci8030050

**Published:** 2021-03-17

**Authors:** Giacomo Galdiero, Emanuele D’Anza, Cristina de Angelis, Sara Albarella, Vincenzo Peretti, Rosario Pivonello, Francesca Ciotola

**Affiliations:** 1 Department of Veterinary Medicine and Animal Productions, University of Naples Federico II, Via Delpino 1, CAP, 80137 Naples, Italy; giacomo.galdiero@unina.it (G.G.); emanuele.danza@unina.it (E.D.); vincenzo.peretti@unina.it (V.P.); francesca.ciotola@unina.it (F.C.); 2 Department of Clinical Medicine and Surgery, Division of Endocrinology, Unity of Andrology and Medicine of Reproduction and Male and Female Sexuality (FERTISEXCARES), University of Naples Federico II, CAP, 80131 Naples, Italy; cristinadeangelis83@hotmail.it (C.d.A.); rosario.pivonello@unina.it (R.P.)

**Keywords:** dog semen, epigenetic, sperm quality parameters, spermatozoa

## Abstract

Male infertility is an emerging problem in both humans and animals, and the knowledge of its causes is the first step to identifying new diagnostic and therapeutic strategies. In humans, alteration of sperm DNA methylation have been related to poor quality semen, impaired seminal parameters, azoospermia and reduced fertility. Although semen analysis is routinely used to evaluate the male reproductive potential in the canine species, no authors have attempted to relate semen characteristics to the sperm global DNA methylation (SGDM). The aim of this study was to evaluate the SGDM level in healthy dogs and to correlate it with semen parameters that are currently used in dog semen analyses. Conventional and unconventional (sperm DNA fragmentation and SGDM) seminal parameters of thirty dogs from different breeds were evaluated. A positive correlation was found between SGDM and sperm concentration (r = 0.41; *p* < 0.05), and total sperm count (r = 0.61; *p* < 0.001); SGDM was significantly lower in oligozoospermic vs non-oligozoospermic dogs (4.3% vs. 8.7%; *p* < 0.005). Our findings suggest that SGDM levels are related to conventional seminal parameters, and could be used as a marker of testis function and spermatogenesis in dogs.

## 1. Introduction

Reproduction and fertility are the most important traits in domestic animals; thus, a lot of studies are aimed at identifying the causes of reproduction failure in domestic species. Among the most investigated fields there are the disorders of sex development [1,2,3,4,5,6] and reproductive performance [7,8,9,10,11,12]. As regards dog species, the occurrence of male infertility is an emerging problem and the knowledge of its causes is the first step to identifying new diagnostic and therapeutic strategies. To date, the main analyses used to evaluate the semen in domestic animals are divided into qualitative (semen volume, aspect, viscosity and pH) and quantitative (sperm concentration/count, motility, morphology, vitality, DNA fragmentation, morphometric) [13,14,15]. However, it has been observed that in humans also when these parameters are normal, semen may not be fertile, and it has been proven that sperm global DNA methylation (SGDM) is related to human fertility rate also independently from other seminal parameters, such as sperm count, progressive motility and morphology [16]. Early evidence for a link between epigenetic markers and male fertility was provided by studies in rodent models, which revealed that exposure to 5-azacytidine triggers a dose-dependent DNA hypomethylation in spermatozoa [17]. Moreover, rats treated with 5-aza-2′-deoxycytidine showed impaired testicular histology, reduced sperm counts and infertility [18]. DNA methylation is an epigenetic modification consisting in changes, sometimes heritable, influencing gene expression that do not cause changes in DNA sequence [19]. Epigenetic changes not only have an impact on developmental processes and fetal growth [20], but are also relevant in many different areas of biology and medicine, including cancer, aging and environmental toxicology [21,22,23,24]. As regards spermatogenesis, epigenetic changes are involved in the proper arrangement and maintenance of the sperm genome and exert crucial effects on sperm quality and function and fertilization potential. A complex and precise epigenetic reprogramming takes place starting from germ cells during migration to the genital ridge and is essential for spermatogenesis completion [25,26]. In humans, alteration of both SGDM and gene-specific (i.e., H19, MEST, BRDT, MTHFR) DNA methylation patterns have been related to poor quality semen, impaired seminal parameters, azoospermia and reduced fertility [27,28,29,30,31,32]. The first association between methylation levels and infertility was reported by Benchaib et al. [33], who demonstrated that SGDM levels above an arbitrary threshold were seemingly linked to high pregnancy rates, suggesting that SGDM status independently affects embryogenesis. Urdinguio et al. [16] showed significant differences in SGDM levels between fertile and unexplained infertile patients, with significantly lower methylation levels in spermatozoa from infertile individuals. El Hajj et al. [34] found a significantly lower methylation level in semen samples resulting in abortions, compared to those leading to a delivery. Independently from the cause and the time of occurrence of the epigenetic alterations (in utero vs prepubertal vs adulthood), it has been demonstrated that the sperm epigenetic landscape has transgenerational effects and is likely influential in the developing embryo. Indeed, mature sperm provide epigenetic marks that drive the activation/inactivation of specific genes by contributing to the pluripotency of the embryonic cells and by influencing its future adult health status, including fertility and reproductive disorders [29,32,35]. All these studies support the hypothesis that the sperm DNA methylation pattern of both imprinted and non-imprinted genes is essential for normal sperm function, fertility and embryo development. An improved knowledge of sperm epigenetics is not only necessary to understand the physiology of reproduction, but also to provide clues on the potential causes of male infertility of unknown origin. The owned dog is considered as an advantageous model of study for human biology and disease [36]; in fact, it generally cohabitates with its human owner, thus reducing differences due to environmental effects, and it receives medical care almost like a human [37]. The aim of this study was to evaluate the SGDM level in healthy dogs and to correlate it with conventional and unconventional semen parameters used in semen analysis of this species.

## 2. Materials and Methods

### 2.1. Sampling and Evaluation of Semen Quality

Thirty dogs from different breeds, clinically healthy, with normal weight considering the breed, and with at least one litter in the latter 12 months were involved in this study (Table 1). To exclude external factors that could alter DNA methylation, only dogs housed under standard conditions and not taking drugs were included in the study. After three days of abstinence, obtained by a previous controlled manual manipulation, the semen was collected by manual stimulation as described by Kutzler [38]. The first and second fractions of the ejaculate were collected in the same tube, while the third fraction was collected in another tube using glass funnels. Semen quality was evaluated in the combined first and second fractions. Volume (mL) was determined using a graduated tube. Sperm count was evaluated with the Makler Counting Chamber; sperm motility was visually assessed under a phase contrast microscope (Nikon Eclipse 80i) at ×200 magnification; sperm morphology was evaluated at the optical microscope (×1000) by Giemsa staining. Sperm concentration (×10⁶/mL), followed by calculation of the total sperm count (sperm concentration × semen volume), and percentage of motile spermatozoa (%) were determined according to the WHO (World Health Organization) guidelines and procedures by classifying spermatozoa motility in progressive motility, in situ sperm motility and immotility [39]. Dogs are defined normozospermic when the sperm count is greater than 300 × 10^6^, the percentage of progressively motile spermatozoa is 70% or greater and the percentage of morphologically normal spermatozoa is 60% or greater [40]. Dogs are defined oligozoospermic when the sperm count is <300 million and non oligozoospermic when the sperm count is greater than 300 million.

### 2.2. Head Area Analysis

Among morphometric parameters, sperm head area is of great interest because it has been related to fertility parameters [41,42,43,44]. It is, in fact, an index of chromatin condensation. At least 200 spermatozoa (50 per slide) from each ejaculate were observed in a bright field under a Nikon Eclipse 80i microscope (100×), captured with a digital camera (Nikon DS-Ri1) and analyzed with the software Nis Elements Imaging Software 4.00.02 (Nikon, Tokyo, Japan) for head area measurement.

### 2.3. Leukocytes Depletion and Spermatozoa Purification

Semen samples were processed with Dynabeads^®^ CD45 magnetic beads (Invitrogen, Carlsbad, CA, USA) in order to obtain purified spermatozoa samples, free from contaminating leukocytes. Thereafter, spermatozoa were separated from somatic cells using discontinuous two-layer (40:80: vol./vol.) density gradient (PureSperm) (Nidacon International AB, Molndal, Sweden). After centrifugation for 30 min at 300× *g*, the spermatozoa pellet was collected and washed twice with phosphate-buffered saline (PBS). Purified spermatozoa were subsequently used for DNA extraction.

### 2.4. DNA Extraction

Each spermatozoa sample was washed twice in 10 mL of Wash Buffer containing 150 mM NaCl and 10 mM EDTA (ph 8.0) in DNAse and RNAse free water, and centrifuged at 750× *g* for 10 min. Subsequently, samples were incubated for 2 h in water bath at 56 °C in DNA Extraction Buffer (4,24 M guanidine thiocyanate, 100 mM NaCl, 1% Sarkosyl, 150 mM DTT and 200 μg/mL proteinase K in DNAse and RNAse free water). DNA was precipitated in isopropanol, spooled with a U-shaped Pasteur pipette and transferred to a tube containing Sodium Citrate in 10% EtOH. DNA was then washed twice in 70% EtOH. DNA samples were resuspended in 500 µL of 10 mM Tris-HCl pH 8.0 and stored at 4 °C until use [45].

### 2.5. 5-mC DNA ELISA

SGDM was evaluated by using an EZ DNA Methylation™ Kit (Zymo research, Irvine, CA, USA), according to the manufacturer instructions. Briefly, the percentage of 5-Methylcytosine (% 5-mc), a surrogate marker of SGDM, was evaluated in each DNA sample by loading 100 ng of denatured, single-stranded DNA in a 96-well plate coated with an anti-5-mc monoclonal antibody and the HRP-conjugated secondary antibody. Detection of % 5-mc was performed after addition of the HRP developer and quantitation was performed by reading absorbance at 405–450 nm using an ELISA plate reader and the logarithmic equation of the line from the standard curve that was constructed with negative and positive controls and standards with known % 5-mc. Each DNA sample was assessed in duplicate.

### 2.6. Determination of DNA Integrity

Sperm DNA fragmentation (SDF) was evaluated by Halosperm^®^ kit (Halotech^®^ DNA SL, Madrid, Spain), according to manufacturer instructions. The slide was left to dry at room temperature and therefore stained for direct microscopic observation under light microscopy. A minimum of 500 spermatozoa per sample were analyzed and scored.

### 2.7. Statistical Analysis

Results were analyzed using IBM SPSS for Windows software package version 22.0 (SPSS Inc., Chicago, IL USA). Dogs were divided in two groups according to their size: medium (<25 kg) and large (<45 kg). This classification was performed since size affects semen volume and total sperm count [46]. Groups were analyzed separately and then grouped two by two (medium and large). Skewness test, Kurtosis test, Z value and Shapiro test were performed to verify parameters distribution (parametric/non-parametric). Data are presented as medians and interquartile (IQRs). The independent-sample *t*-test (Mann–Whitney test) was used to compare medians of quantitative variables. Spearman’s test was used to assess correlation between SGDM level and other seminal parameters. Statistical significance was set at *p* ≤ 0.05.

## 3. Results

**Figure 1:**
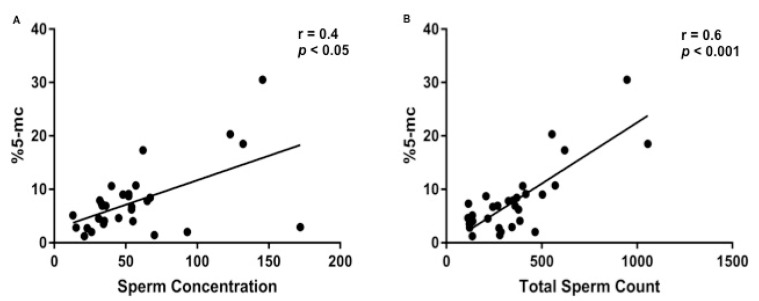
Relationship between %5-mc and sperm concentration (**A**) and total sperm count (**B**) in dogs population. Spearman’s correlation analysis shows a significant regular positive correlation between %5-mc and sperm concentration (r = 0.4; *p* < 0.05) (**A**) and a significant strong positive correlation between %5-mc and total sperm count (r = 0.6; *p* < 0.001) (**B**).

**Figure 2:**
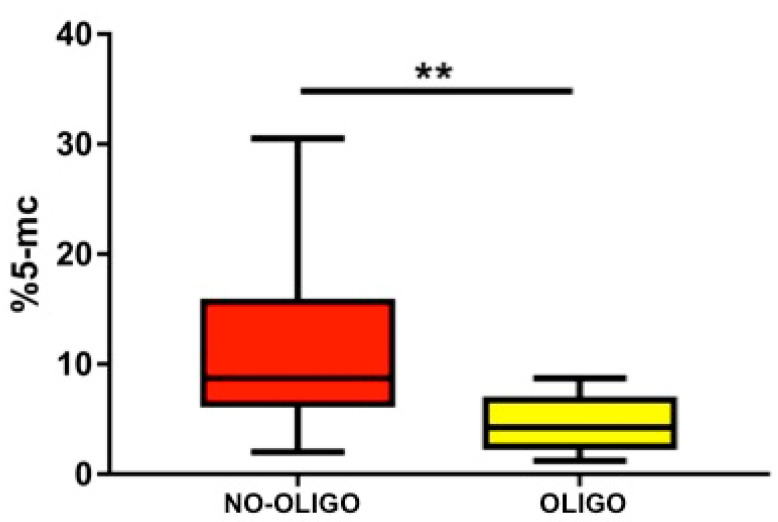
The box plot shows the sperm global DNA methylation (SGDM) (%5-mc) in non oligozooospermic/oligozoospermic dogs, in the total study population. NO-OLIGO: Non oligozoospermic; OLIGO: Oligozoospermic. A significant difference was found ** *p* < 0.005.

**Figure 3:**
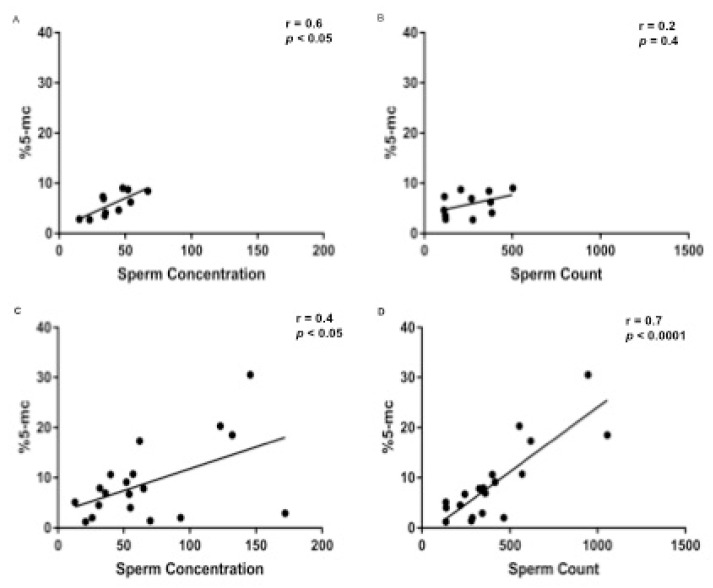
Relationship between %5-mc and sperm concentration (**A**,**C**) and total sperm count (**B**,**D**) dividing the samples according to dog’s size: medium sized (**A**,**B**) and large sized (**C**,**D**). Spearman’s correlation analysis shows a significant strong positive correlation between %5-mc and sperm concentration in medium sized dogs (r = 0.6; *p* < 0.05) (**A**) and large sized dogs (r = 0.4; *p* < 0.05) (**C**). A significant strong positive correlation between %5-mc and total sperm count was found in large sized dogs (r = 0.7; *p* < 0.0001) (**D**).

**Figure 4:**
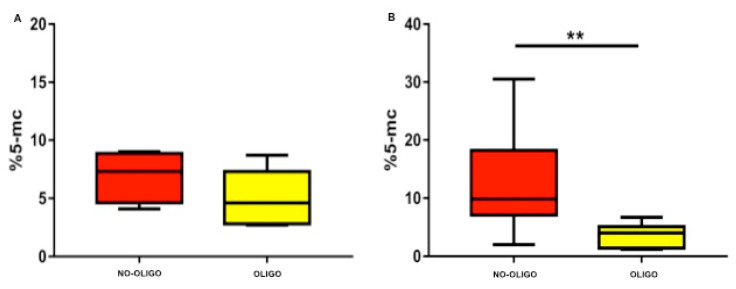
Histogram showing the SGDM (%5-mc) in non oligozooospermic/oligozoospermic dogs, dividing the samples according to dog’s size. NO-OLIGIO: Non oligozoospermic; OLIGO: Oligozoospermic. Medium sized dogs (**A**) show no significant differences. Large sized dogs (**B**) show a significant difference (** *p* < 0.005).

Median and IQR (5°–95° percentile) body weight and age of the analysed dogs were 40 (15–60) kg and 3 (1.3–8.5) years, respectively. Median and IQR values for qualitative and quantitative seminal parameters were as follow: semen volume 7 (2.3–11.5) mL; sperm concentration 50 (14.2–157.5) × 10⁶/mL; total sperm count 334.5 (114.2–996.1) × 10^6^/ejaculate; progressive sperm motility 75 (25.5–95) %; normal sperm morphology 76 (50–82)%; sperm head area 17.1 (12.9–18.9) μm²; SDF 2.6 (0.9–8.7) % (Table S1). Prevalence of oligozoospermia, asthenozoospermia and teratozoospermia were 46% (14/30), 34% (10/29) and 20% (6/29), respectively. When evaluating SGDM we found a value of 6.8 (1.3–24.9) %, ranging from 1.2 and 30.5% (Table S1). When analyzing data dividing the dogs according to their size (medium and large) a statistical difference was observed in body weight (*p* < 0.0001), as expected, and in total sperm count (*p* < 0.05) (Table S2). The Skewness test, Kurtosis test and Z value and Shapiro test showed that data were non-parametric distributed. According to Spearman’s rank coefficient (Table S3), a regular positive correlation was found between body weight and sperm concentration (r = 0.5; *p* < 0.005) and between body weight and total sperm count (r = 0.6; *p* < 0.05) (Figure S1) while a regular positive correlation and a strong positive correlation were found between SGDM percentage and sperm concentration (r = 0.4; *p* < 0.05), and total sperm count (r = 0.6; *p* < 0.001), respectively (Figure 1). SGDM percentage was significantly lower in oligozoospermic dogs, when compared to those with total sperm counts above the normality threshold (4.3% vs. 8.7%; *p* < 0.005) (Figure 2), whereas no significant difference was found in SGDM percentage when stratifying animals based on progressive sperm motility or sperm morphology normality thresholds. A further analysis, performed dividing the samples according to dog’s size (medium and large), showed a strong positive correlation between SGDM percentage and sperm concentration in medium (*n* = 11) and large (*n* = 19) sized dogs (r = 0.6, *p* < 0.05; r = 0.4, *p* < 0.05) and a strong positive correlation between SGDM percentage and total sperm count in large (*n* = 19) sized dogs (r = 0.7, *p* < 0.0001) (Figure 3). Based on the same dog’s size grouping, SGDM was assessed in oligozospemic and non-oligozospemic dogs. The prevalence of oligozoospermia in medium sized and large sized was 63% (7/11) and 36.8% (7/19), respectively. The SGDM percentage was overall lower in oligozoospermic dogs compared to those with total sperm counts above normality threshold in all groups, with a statistically significant difference in large sized dogs (4% vs. 9.8%; *p* < 0.005), but not in medium sized (4.6% vs. 7.2%) (Figure 4).

## 4. Discussion

Defects of SGDM level have been related in humans and in animal models (i.e., rodents) to sperm DNA damage and defective spermatogenesis [18,47]. Houshdaran et al. [48] reported that poor semen quality samples displayed an abnormal global DNA sperm hyper-methylation, as assessed by the analysis of repetitive elements, and locus-specific sperm DNA at imprinted and non-imprinted genes. Conversely, the majority of studies showed an abnormally reduced SGDM in poor semen quality samples. Most studies demonstrated that SGDM level is associated not only with sperm concentration but also with sperm motility: oligozoospermic and severely asthenozoospermic men have significantly lower levels of SGDM, compared to normozoospermic men or men with moderately impaired motility [35,47,49].

Sperm Global DNA hypomethylation, in particular, is associated with fertility alterations in humans with normal and abnormal seminal parameters [47]. Up to now no data are available about SGDM in dogs, a species whose breeding is of strong interest, and which at the same time is considered an important study model for human with which it often shares domestic environment, lifestyle and exposure to pollutants. The first main data were the absence of statistical differences among average age of the dogs when grouped according to their size, indicating that the groups were homogeneous by age. This prevented that aging could impact the differences eventually observed among groups in SGDM percentage (Table S1) [50]. Data reported in this study showed a relationship between dog size and sperm concentration and total sperm count; thus, the larger the dog is, the higher is the number of produced spermatozoa. As regards sperm head area, no significant differences were found among dogs grouped according to their size, and values of this parameter ranged between 12.2 and 19.4 μm². The relationship between dog size and sperm concentration and total sperm count is in line with previous observation and it is due to the higher volume of testes of the larger dogs [51]. Moreover, this study showed, for the first time, a relationship between SGDM measured as % 5-mc and seminal parameters in healthy dogs which had fathered at least one litter. According to statistical analysis overall levels of SGDM correlated positively to sperm concentration and total sperm count, both in the general population and in dogs grouped according to size. SGDM was significantly lower in oligozoospermic dogs, compared to those with total sperm counts above the normality threshold (>300 × 10^3^), in the general population, as well as in both the group of medium and large sized dogs. SGDM percentage was lower in oligozoospermic vs non-oligozoospermic medium sized dogs, although this difference was not statistically significant, probably due to small sample size of the two groups; in fact, in the group of large dogs which had double the number of dogs, SGDM was significantly different between oligozoospermic vs non-oligozoospermic dogs, with oligozoospermic dogs displaying a significantly lower SGDM percentage.

These results suggest that epigenetic changes, specifically SGDM, might be used as a marker of testis function and spermatogenesis in dogs. It can be hypothesized that higher SGDM levels might correspond to improved spermatogenesis. This is in line with data reported in humans, showing a lower level of SGDM in oligozoospermic men [16,30,33,34,35,47,52]. The positive correlation, found in this study, between SGDM and sperm concentration and total sperm count suggest that improper DNA methylation might be associated with spermatogenesis alterations that reduce the number of spermatozoa. This is also confirmed by the differences in methylation percentage observed between oligozoospermic and non-oligozoospermic.

## 5. Conclusions

To the authors’ knowledge, this is the first report in which a correlation of SGDM percentage with conventional semen parameters in dogs is shown. The finding of a similar correlation between methylation levels and seminal parameters in humans and dogs shows that the latter can be used as a valid animal model for sperm methylation studies. In particular, it would be interesting to verify also in this species the correlation between SGDM percentage and outcome of assisted reproduction techniques, recurrent pregnancy loss, etc. Finally, future studies should be aimed at evaluating the relationship between SGDM and other factors potentially affecting dog semen quality and fertility, to verify if it could be used as further analysis to be added to conventional seminal parameters in order to increase their predictive value for the dog reproductive performances. Moreover, considering that aberrant DNA methylation and chromatin compaction may result in inherited genomic errors, a wider implication of study results might comprise the role of epigenetic changes in congenital diseases affecting the offspring.

## Figures and Tables

**Table 1:** Breed and age of the analyzed dogs. (Y = year).

Breed	N° of Animals	Age Range (Y)
Neapolitan Mastiff	8	2–4
German shepherd	5	1–6
English Bull dog	4	2–5
Dachshund	2	2–9
Beagle	1	8
Caucasian Shepherd dog	1	4
Maremmana Sheepdog	1	5
Pointer	1	3
Kangal	1	2
Labrador retriever	1	2
Half-breed	5	2–8

## Data Availability

The data that support the findings of this study are available from the corresponding author upon reasonable request.

## Supplementary Materials

The following are available online at https://www.mdpi.com/2306-7381/8/3/50/s1, Figure S1: Relationship between body weight and sperm concentration, Table S1: Dogs semen quality and quantity parameters, Table S2: Dogs semen quality and quantity parameters by dividing the dogs according to their size (Medium and large), Table S3: Spearman’s rank coefficients.
