# Supplementary material for: Sperm Global DNA Methylation (SGDM) in Semen of Healthy Dogs
Source: Vet Sci. 2021 Mar 17;8(3):50. doi: 10.3390/vetsci8030050 (PMC8002840; doi:10.3390/vetsci8030050)
Supplement: Supplementary file 1 [file vetsci-08-00050-s001.pdf]

Supplementary Materials:

Table S1. *Dogs semen quality and quantity parameters.*

PARAMETER	MEDIAN (5°-95° IQR)	N°
Body weight (kg)	40 (15-60)	30
Age (years)	3 (1.3-8.5)	30
Semen volume (ml)	7 (2.3-11.5)	30
Sperm concentration (x10^6/ml)	50 (14.2-157.5)	30
Total sperm count (x10^6/ejaculate)	334.5 (114.2-996.1)	30
Progressive sperm motility (%)	75 (25.5-95)	29
Normal sperm morphology (%)	76 (50-82)	29
Sperm head area (μm^2)	17.1 (12.9-18.9)	30
SDF (%)	2.6 (0.9-8.7)	12
SGDM (%)	6.8 (1.3-24.9)	30

Table S2. *Dogs semen quality and quantity parameters by dividing the dogs according to their size (Medium and large)*

	MEDIAN (5°-95°IQR)		
**PARAMETER**	**Medium sized**	**Large sized**	**P**
Body weight (kg)	25 (15-35)	60(35-60)	<0.0001
Age (years)	3 (1.5-8)	3 (1-9)	NS
Semen volume (ml)	7 (2.5-12)	7 (2-11)	NS
Sperm concentration (x10^6/ml)	35 (15.3-67)	55 (13-172)	NS
Total sperm count (x10^6/ejaculate)	268.8 (112.5-504)	349.8 (136.5-1056)	<0.05
Progressive sperm motility (%)	74.3 (47.6-93.2)	78 (21-95)	NS
Normal sperm morphology (%)	74.5 (50-80)	78 (50-84)	NS
Sperm head area (μm^2)	17.3 (13.6-18.2)	16.9 (12.2-19.4)	NS
SDF (%)	2 (1-8.7)	2.8 (0.9-4.6)	NS
SGDM (%)	6.2 (2.7-9)	6.9 (1.2-30.5)	NS

Table S3. *Spearman's rank coefficients.*

Negative	Level	Positive
[-1]	Perfect	[-1]
[-0.9 to -1]	Very Strong	[0.9 to 1]
[-0.6 to -0.9]	Strong	[0.6 to 0.9]
[-0.3 to -0.6]	Regular	[0.3 to 0.6]
[0 to -0.3]	Weak	[0 to 0.3]
[0]	Absent	[0]

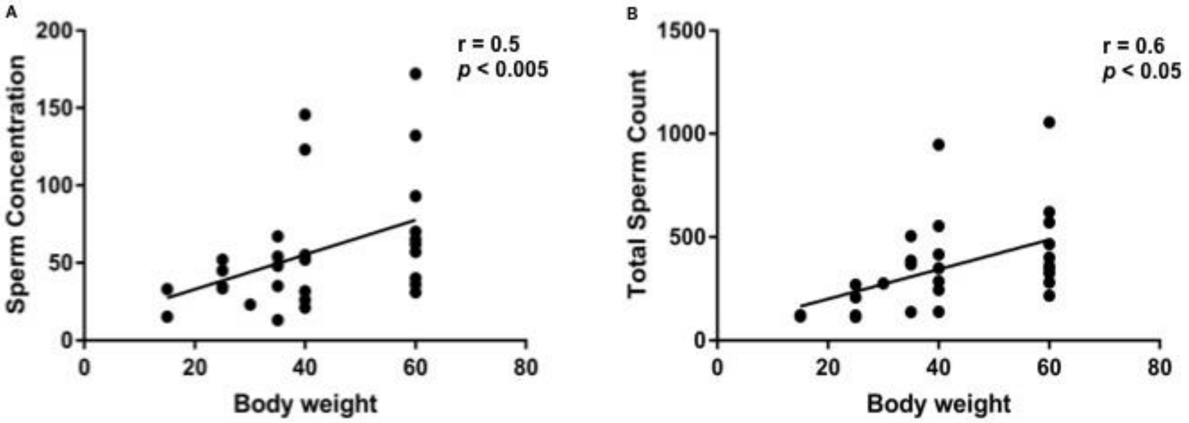

Figure S1. *Relationship between body weight and sperm concentration*
